# Case of Hydrothorax Successfully Treated

**Published:** 1813-05

**Authors:** Arnall Thomas Fayerman

**Affiliations:** Surgeon, Fellow of the London Medical Society, and late Lecturer on Chemistry to the Harleston Philosophical Institution. No. 22, Orford Hill, Norwich


*To the Editors of the Medical and Physical Journal.*

GENTLEMEN,

PERHAPS there is no disease to which the human subject is liable, so fatal in its consequences, or so distressing to the patient, and treated so variously (generally unsuccessfully) as Hydrothorax, or Dropsy of the Chest. I consider it a duty incumbent on every medical man, whose diligent attention to his profession enables him to define the cause and nature of disease, and method of cure, to publish every case of successful practice, for the benefit of the medical world, and society in general. Obedient to this suggestion, I beg leave, through the medium of your respectable Journal, to transmit the *fourth* case which has occurred to me of Hydrothorax within the period of three years, where *complete* recovery has been the consequence of the plan of treatment which I have adopted.

I have the honor to be, Gentlemen,

With much respect, your obedient Servant,

ARNALL THOMAS FAYERMAN, Surgeon,
Fellow of the London Medical Society, and late Lecturer on Chemistry to the Harleston Philosophical Institution.
No. 22, *Orford Hill, Norwich,*
*March* 14, 1813.

On Wednesday morning, January the 13th, 1813, I was called to attend Mr. Metcalfe, a person of robust habit, ætat. 54, a respectable shawl manufacturer, in the parish of St. Simon's, in this city. I found him in a reclining position on the back of a chair, the only attitude he could place himself in for relief. On taking an accurate inspection of his appearance as I entered the room, it gave me an instant idea of the disease under which he was laboring, namely, Hydrothorax. This opinion was fully confirmed on making a few inquiries. He informed me, that, about three years preceding the period I was called on to attend him, he had complained of much fatigue and lassitude after any particular exercise, a sense of tightness and weight across the sternum, a teasing cough, increased on lying down, occasional loss of appetite, costive stools, and stoppage of urine. On putting a few questions as to his manner of living, he candidly confessed that he had habituated himself to the use of strong malt liquors to great excess. The symptoms abovenamed had continued to increase slowly upon him, gradually undermining his strength, becoming every succeeding day more aggravated and alarming. About the 8th of June last, œdematous swellings of the legs and ancles came on; the rest at night disturbed by frequent spasms, threatening immediate suffocation, so much, to use his own words, "as to occasion me to throw myself out of bed as quick as possible, and get to the window for air." About this time, June the 14th, medical advice was desired by his friends, and acceded to by himself; however, notwithstanding the various means resorted to for his recovery by the professional gentlemen in attendance, the disease continued to advance with hasty strides; and he became, about the beginning of October, totally unable to lie in a horizontal position. Even in a recumbent posture, with the legs elevated, the spasms instantly returned, so as to oblige him to get up from the chair and walk about. The following are the symptoms which marked the case on the morning of the 13th of January, 1813: An appearance of great grief and anxiety strongly depicted in the countenance; great depression of spirits; drowsy stupor; dilated pupils of the eyes; breathing laborious and short; dull sense of pain, and great oppression in the chest; anxiety about the pericardium; the pulse slow, full, and intermitting; the legs and ancles swelled enormously; the cuticle of the right leg had burst, and the water was running from it; the stools costive, and of a clayey color; the urine small in quantity, and very pale.

Notwithstanding the above very formidable evils which presented themselves to common observers, (and indeed to two medical men who had previously seen him, and given up the case as hopeless,) I did not feel dismayed, being confident, that if I could not complete a cure, I could at least, from former experience, greatly relieve the most distressing symptoms. On suggesting this to his wife and relatives, and that I thought the means I was about to put in execution would in a week enable him to lie down in his bed, I received (as might have been expected) only an incredulous smile, and significant shake of the head. On the same evening I applied a large blister over the sternum; one drachm of the Ung. Hydrarg. fort. was directed to be rubbed inside of each leg, from the ancle to the knee, for half an hour.

Thursday, January 14th.—Found the patient in the same position as the day preceding. The unguent had been duly applied. The blister had performed its operation well; a plentiful discharge had been produced. The blister was removed in the evening, and the following dressing applied:

℞. Ung. Resin. flav. ℥i.

Pulv. Lyttæ ij. M. ft. Ung. pro Epispast.

Ordered

Ordered the following night draught:

℞. Æther Sulph.

Tinct. Castor ā ʒi.

——— Digitalis gtt. xxv.

Mist. Camphor ℥i M. ft.

Haust hora somni sumendus.

Friday, 15th.—Copious discharge from the blister. The pulse not so full, and intermitting. Breathing rather relieved. Cough very troublesome. Legs much swelled. Prescribed the following medicine:

℞. Infus. Digitalis ℥ij. (viz. ℥fs Fol. Digit. purp. to ℔j. Aq. Ferventis.)

Æther Sulph.

Tinct. Galban.

——— Castor ā ʒi.

——— Opii Camph. ʒifs.

Aq. Puræ ℥v. M. ft.

Mist. capiat cochl. amp. iij. xj matutine et hor. 4 vesp. quotid.

℞. Oxyd. Zinc. gr. xviij.
Gum Ammon.
Pilul. Galban. C.
Ext. Cicutæ ā gr. j.
Pulv. Scillæ gr. ix.
ft. Pil. xviij. sumat. iij cum sinq. dos. Mist.

Mercurial frictions repeated at night, and a dose of the following pills at bed-time:

℞. Hydrarg. Submur.
Pulv. Digitalis.
Pulv. Scillæ ā gr. iij.
Sulphat. Zinc. gr. ifs.
Camphoræ gr. ix.
Pilul. Galban. C. gr. xv.
Ol. Junip. gtt. vj. M. ft. Massa et divid. in Pilul. No. ix. Capiat iij. omni nocte hora Somni.

Saturday, 16th.—Visited the patient about noon. Agreeably surprised to find the swelled legs reduced in size. Breathing more easy. Cough still very troublesome. Profuse discharge from the blister. Bowels tolerably regular. Urine more copious, and depositing a lateritious sediment. Pulse more regular, and soft. Mercurial frictions repeated.

Sunday, 17th.—Found the patient in bed when I called, about 10, a. m. Had slept quietly about an hour and a half in the night. The spasms on lying down not so frequent, and less severe. Breathing much easier. Pain and fulness in the chest greatly abated. Pulse regular, and soft. The swelling in the right leg much reduced. Cough frequent,

 and and occasionally severe. Bowels regular. The quantity of urine made during the night near three pints, depositing on standing for a few hours a lateritious sediment. Mercurial frictions repeated.

Monday, 18th.—The swelling of the legs so much reduced as to enable the patient to button his small-clothes at the knees, which he had not accomplished for many weeks. The blister still kept open, and the discharge from it plentiful. A very slight degree of pain and soreness in the chest. Breathing easier. The cough, however, at intervals, very severe; no expectoration with it. Pulse about 80, soft, and without intermission. The urine made during the day and night seven pints and a half, and much clearer. Bowels regular. Slept two hours in the night. Spasms less severe. The day and night medicine repeated, and the mercurial frictions also.

Tuesday, 19th.—Still continuing to advance in recovery; the night passed in ease and quiet; about three hours sleep procured at intervals. A recurrence of spasm on coughing, but very slight. The countenance wonderfully changed for the better. The unnatural dilatation of the pupils of the eyes no longer existing. Pulse softer, and at 80. Occasional slight pain in the chest rather increased this day. Bowels regular. The blister discharging less, and disposed to heal. Legs greatly reduced. The urine made this day and night about one gallon, of a red color, without any deposition of sediment. Mercurial frictions repeated.

Wednesday, 20th.—Convalescence strongly observable. The preceding night passed with six hours sleep at intervals. No return of spasm. Pain in the chest less. Breathing much improved. Appetite good. Bowels regular. Urine copious, and without sediment. Cough better. The blister discharging less, and dressed with the Ung. Sp. Cet. The legs much reduced, and flaccid to the touch. The alterative day medicine and mercurial frictions repeated.

Thursday, 21st.—Continuing to improve. Night passed well. The breathing more regular, and easy. Cough better. No recurrence of spasm on lying in the horizontal position. The stools loose, and of good color. Pulse soft, and without intermission, and about 75. Blister gradually healing. Urine made during the day and night six pints and a quarter. Appetite good. Legs decreasing in size. Night pills and the mercurial frictions repeated.

Friday, 22d.—Appearances much the same as the preceding day. The night good. Appetite good. Bowels regular. Urine about four pints in the day and night, clear, and and without sediment. No pain felt in the chest. No spasms. Pulse soft, and at 70. The mercurial frictions repeated.

Saturday, 23d.—The day being remarkably fine and clear, the patient was induced, in consequence of his improved state of health, to undertake a short walk, which he accomplished without much fatigue. Every symptom of the disease was this day much lessened. Slept five hours without intermission. Spasms entirely gone. Pain in the chest quite removed. Appetite good. Bowels regular. Blister healed. Urine about three pints in the night and day. The legs nearly restored to their natural size. Pulse 75. Cough occasionally returning, but much less severe. The day medicine and mercurial frictions repeated.

Sunday, 24th.—Continuing to improve. Night good. Appetite good. Bowels regular. Pulse 70. Urine about three pints in day and night. Cough better. Night pills and mercurial frictions repeated.

Monday, 25th.—Slight giddiness and pain in the head felt. Pulse at 70. Urine more copious. Complained of being rather sick. Appetite not quite so good. Bowels rather relaxed. Legs much reduced. No cough or spasms. The preceding night passed with ease. The Infus. Digit. given in less quantity, viz. ℥i to ℥v of the aforesaid menstruum, thinking it the cause of sickness and head-ach. Mercurial frictions repeated.

Tuesday, 26th.—Found the patient to-day much better. Walked out about a quarter of a mile and back; fatigue but slight. Appetite good. Bowels not so much relaxed. Sickness removed. Pain of the head gone. Rest at night undisturbed. Urine about three pints.

Wednesday, 27th.—The day medicine persisted in. Every symptom of hydrothorax gone, and a rapid convalescence establishing. The appetite good. The night passed in uninterrupted serenity. The legs restored to their natural size. Bowels regular. Pulse 75. Urine clear, and much in quantity as usual. Cough gone.

Thursday, 28th.—Two ounces and one drachm of strong mercurial ointment had now been rubbed into the legs, and no symptom of ptyalism had made its appearance. A mixture of the Decoct. Cinchonæ and Infus. Digitalis with Æther, was this day substituted for the former. The night pills continued, and the mercurial frictions also.

From the 28th of January to the 1st of March, the medical plan was followed up, accompanied with the mercurial frictions.

*Remarks.*—A circumstance occurred in the earlier part of the treatment of this disease, which perhaps it may be worth while to record. The nurse, by mistake, one evening, rubbed in the Ung. pro Epispast. instead of the Ung. Hyd. fort. A plentiful eruption was thrown out on the surface of the legs, which were also much inflamed, the consequence of which most probably hastened the reduction of the œdematous swelling. Mr. Metcalfe is now enabled to walk two or three miles without much fatigue, and follows a laborious profession. In fact, he says his health is now much better than it has been for many years.

